# Supporting the classification of patients in public hospitals in Chile by designing, deploying and validating a system based on natural language processing

**DOI:** 10.1186/s12911-021-01565-z

**Published:** 2021-07-01

**Authors:** Jocelyn Dunstan, Fabián Villena, Jorge Pérez, René Lagos

**Affiliations:** 1 Center for Mathematical Modeling - CNRS UMI2807, Faculty of Physical and Mathematical Sciences, University of Chile, Santiago, Chile; 2 Center for Medical Informatics and Telemedicine, ICBM, Faculty of Medicine, University of Chile, Santiago, Chile; 3 Computer Science Department, Faculty of Physical and Mathematical Sciences, University of Chile, Santiago, Chile; 4 Millennium Institute for Foundational Research on Data, Santiago, Chile; 5 Digital Health Unit, South East Metropolitan Health Service, Santiago, Chile

**Keywords:** Decision support systems, Waiting lists, Natural Language processing, Machine learning, Neural networks (computer)

## Abstract

**Background:**

In Chile, a patient needing a specialty consultation or surgery has to first be referred by a general practitioner, then placed on a waiting list. The Explicit Health Guarantees (GES in Spanish) ensures, by law, the maximum time to solve 85 health problems. Usually, a health professional manually verifies if each referral, written in natural language, corresponds or not to a GES-covered disease. An error in this classification is catastrophic for patients, as it puts them on a non-prioritized waiting list, characterized by prolonged waiting times.

**Methods:**

To support the manual process, we developed and deployed a system that automatically classifies referrals as GES-covered or not using historical data. Our system is based on word embeddings specially trained for clinical text produced in Chile. We used a vector representation of the reason for referral and patient's age as features for training machine learning models using human-labeled historical data. We constructed a ground truth dataset combining classifications made by three healthcare experts, which was used to validate our results.

**Results:**

The best performing model over ground truth reached an AUC score of 0.94, with a weighted F1-score of 0.85 (0.87 in precision and 0.86 in recall). During seven months of continuous and voluntary use, the system has amended 87 patient misclassifications.

**Conclusion:**

This system is a result of a collaboration between technical and clinical experts, and the design of the classifier was custom-tailored for a hospital's clinical workflow, which encouraged the voluntary use of the platform. Our solution can be easily expanded across other hospitals since the registry is uniform in Chile.

## Background

The analysis of the clinical free-text is challenging due to the generally non-standardized use of abbreviations and acronyms, the presence of negation, speculation and temporal expressions, or the limited availability of training corpora due to privacy concerns, among others [1]. The challenge is even steeper when working on languages other than English because of the limited availability of tools and training corpora [2]. Even though Spanish is one of the most spoken languages in the world, the number of language resources is still insufficient, especially beyond Spain and the United States [3].

In Chile, the Public Health Fund FONASA covers 74% of the population [4]. In contrast to the private sector, where a patient can go directly to a specialist, patients in the public system need a referral from a general practitioner in primary care, which puts them in the national registry for the waiting list (WL) in the area where they are seeking medical attention. To address health inequalities and health deterioration due to prolonged waiting times, in 2006, the Chilean government implemented the Explicit Health Guarantees (known as GES for its acronym in Spanish). GES prioritizes 80 health conditions and their maximum amount of time, starting from the initial referral time, that patients may have to wait for treatment. It also offers a higher economic coverage than non-GES pathologies for specific pathologies and care services [5]. An example of a GES condition is a cataract, a cloudy area in the eye lens that leads to poor vision. On the other hand, a non-GES example is a glaucoma, which damages the optic nerve and can cause loss of vision.

Public hospitals receive incentives to efficiently deal with GES-covered referrals, leading to dramatic differences in waiting times and volume of WL when one compares GES to non-GES waiting lists [6]. Prolonged waiting times in the non-GES WL have been studied using hierarchical multivariate survival models applied to nearly a million patients, finding a statistically significant association between waiting time and mortality [7]. According to the latest published information [8], 1% (34,305 people) of the patients died in 2019 while waiting in the non-GES WL. These numbers contrast to the 1.6% (830 people) of patients in the GES WL during the same year.

In 2018, the Chilean Healthcare Administration estimated that around 10% of patients with GES diagnoses were not receiving the prioritized treatment. Moreover, that same year, it sanctioned 83 healthcare institutions for the incorrect handling of GES cases [9]. All of these statements imply that proper classification of GES and non-GES referrals is crucial, not only for patients but also for hospitals.

To assess if GES covers a referral, a healthcare professional needs to check if the free-text reason for referral, stated by the general practitioner, corresponds to one of 85 specific health conditions and the age of the patient. This assertion is not always easy as each health problem has a subset of different pathologies covered for a given age range. For example, assume that there is a referral with the diagnosis *colelitiasis* (cholelithiasis) for a 36-year-old patient. This diagnosis should be marked as GES since (a) there is a GES health condition called *Colecistectomía preventiva del cáncer de vesícula en personas de 35 a 49 años*, which covers some diseases of the gallbladder for the age range 35 to 49 years old, and (b) this condition specifies a pathology called *Cálculo de la vesícula biliar sin colecistitis* which, although not explicitly, matches the referred diagnosis in the given age of the patient. These difficulties, together with the absence of standardized ways of defining pathologies, the heavy use of abbreviations, and spelling mistakes, among other reasons, require a health professional dedicated to the GES/non-GES classification in most hospitals across Chile. This professional, typically a nurse with experience in case management, reviews the WL manually, and uploads separated GES and non-GES databases to the National Repository.

### Related work

The topic of classifying referrals or patients using Natural Language Processing (NLP) tools is extensive, especially in English. Two systematic reviews on patient classification methods in radiology [10] and oncology [11] compile an extensive list of commercial tools, rule-based, machine learning, and deep learning approaches, and normalization to ontologies such as ICD or SNOMED-CT, used in information extraction and report classification systems. Also, the work of Roque et al. on cohort selection [12] illustrates another application of NLP tools to the biomedical field.

For the Spanish language, Cotik et al. developed an algorithm based on syntactic analysis, entity recognition and hedges and negations identification for classification of radiology report from Argentina [13, 14], while in Chile, Ramos et al. implemented a support decision-making tool to recommend the classification of ‘cancer’ versus ‘not cancer’ and ‘breast cancer’ versus ‘other cancer’ on patient medical histories using word-embedding and machine learning techniques [15], Figueroa et al. used Support Vector Machines and bigram representation to classify patients according to smoking status reported in clinical narratives [16], and Lecaros et al. examined referrals from the Waiting List Repository to identify the detection of patients with psoriasis [17]. From groups in Spain, we acknowledge the work of Soares et al. (18) on word embeddings, which is a key tool for various clinical NLP tasks. Recent developments on clinical text classification include the work of López-Úbeda et al. using transfer learning [19], Blanco et al. working on multi-label document classification [20], and García-Pablos using transformer-based approaches [21].

The work presented here reports the design and performance of an automatic classifier of referrals trained over Chilean clinical text. We achieved this goal by collecting a clinical corpus and computing neural word embeddings. These embeddings were used in a variety of machine learning models, which were deployed as a web service in one of the biggest hospitals in Chile. Our system achieved a ROC AUC of 0.94 and was continually used for seven months. During this time, the system analyzed 4472 referrals and helped to re-classify 87 cases. Since the WL must be uploaded to a National Repository with a uniform format, this classifier has the potential for use in every hospital in Chile. The word embeddings as well as the ground truth dataset and the codes to reproduce results are shared with the research community.

## Methods

### Data

We considered two datasets in Spanish language, one specific *GES/non-GES dataset* and one *general dataset* of Chilean referrals. The *GES dataset* was obtained from a collaboration with the Digital Health Unit at the South East Metropolitan Health Service (SEMHS) in Santiago, which provides healthcare service to 8.3% of the Chilean population. The SEMHS provided de-identified historical data of both GES and non-GES cases between 2005 and 2018. This dataset contains 2,105,129 cases, from which 375,969 were tagged as GES referrals. The access to SEMHS data, as well as the possibility of piloting the classifier at the Hospital Sótero del Rio, was possible due to a data agreement signed between SEMHS and the institution of the authors.

We obtained the second dataset, that we call the *general dataset*, via Chile's Transparency Law [22]. This law allows any Chilean to request de-identified public documents. Because they do not contain sensitive information, we are allowed to use them in publications. It is composed of non-GES referrals from 23 of the 29 health services in the country for years in the range 2008–2018. That resulted in nearly 11 million referrals. We used this dataset as a training *corpus* for the word embeddings, detailed in the next section.

### Unsupervised learning: word embeddings

A good choice to deal with unstructured narratives are models based on artificial neural networks, which have reached state-of-the-art in several tasks [23]. One of the techniques is word embeddings, which map each word to a real vector in *D* dimensions, with *D* much smaller than the vocabulary size [24, 25]*.* The idea of a word embedding is to assign a dense vector to each word in the vocabulary, and within this smaller dimension space, perform operations on these vectors to test the quality of the representation [26–28].

Word embeddings are obtained by training a single-layer neural network over unannotated corpora. The task that fine-tunes the weights in the network can either predict a word from the context words in sentences of the corpus (*continuous bag of words* method) or predict the context words from a central word (*skip-gram* method). We refer the reader to the work by Mikolov et al*. *[24] for details.

In this work, we computed word embeddings using Word2Vec with the *skip-gram* method [24], with a vector dimension of 300, and all the remaining options as default. Before vectorizing, the text was lowercased and tokenized using the NLTK package [29]. In addition, characters other than alphabetical and punctuation were deleted through regular expressions. Finally, we dropped 156,948 sentences because of duplicated data or blank attributes. The average number of tokens per referral was 5.3.

To obtain a single vector for each referral, we took the weighted average of the vector assigned to each word in the referral, which is a standard practice in Natural Language Processing [30, 31]. The different weights to each word were assigned using Term Frequency-Inverse Document Frequency score (TF-IDF). This score is proportional to the frequency of appearance of a given word within the document (in this case a referral), but it offsets this value by the number of documents in which this word appears in the *corpus* (all the referrals) [28]. As it is constructed, stop words (such as *the, a, in*) score low, and semantically richer words (*cancer, pain*) receive a high score. This process gave us a 300-dimension vector for each referral.

On our testing set (40% of the GES/non-GES historical dataset, described in more details later), three different word embeddings were computed using default hyperparameters except for the embedding dimension set in 300. The AUROC performance was Word2Vec 0.9615, fastText 0.9615, and GloVe 0.9613 (the implementation can be seen at the GitHub repository shared within this publication). Since the performance differences between the three are marginal, we selected Word2Vec for simplicity and speed, both important in this real-world application.

The choice of training corpora for the word embedding construction is not trivial. In particular, for the clinical domain, several authors have explored combinations of general language, biomedical literature, and clinical corpora [32–34]. For the clinical Spanish language there is a lack of language resources, with the few corpora coming predominantly from Spain [18, 35, 36]. In the work presented here, we extrinsically tested the Spanish Billion Word Corpus Embeddings (computed over 2,024,959,560 tokens) [37], the Chilean Biomedical corpus (computed over 67,246,025 tokens) [38], and the general dataset described earlier (computed over 56,079,828 tokens), with the latest showing the best classification performance (see “Results” section).

### Supervised learning: GES classifier

The vector representation of the reason for referral, as well as the patient's age (transformed using min–max scaler), were the inputs to train supervised machine learning models. We tested Support Vector Machines, Random Forest, Logistic Regression, and Multi-Layer Perceptron using the scikit-learn package in Python.

The GES/non-GES historical dataset was split into training and testing, using 40% of the dataset for testing. The training subset was balanced by downsampling the majority class. The best hyperparameters were selected via grid search over the training subset using threefold cross-validation and choosing those that maximized the area under the curve of the receiver operating characteristic (ROC AUC). The ground-truth was an independent unlabelled subset that three different experts labeled. The best hyperparameter-tuned algorithm was trained over the entire training subset and was tested over the testing subset and ground-truth dataset.

The optimized models were compared based on their ROC AUC using tenfold cross-validation. We assessed the statistical significance of the difference in performance between the averages calculating the paired t-student test with a Bonferroni *p* value correction.

The best hyperparameter-tuned algorithm was trained over the training subset and was tested over the testing subset and ground-truth dataset, which is described in the next section.

### Ground truth construction and validation

For the creation of a ground-truth dataset, three experts labeled an independent dataset of 942 referrals as GES or non-GES. In the case of discrepancies, the first author decided on the label based on the information contained in the official documents of the Healthcare Superintendence.

This ground truth was used to assess the performance of the best model and to compare the level of agreement between humans. Later an error analysis was conducted to understand the model mistakes over the ground-truth.

### Classification effectiveness and deployment

**Fig. 1:**
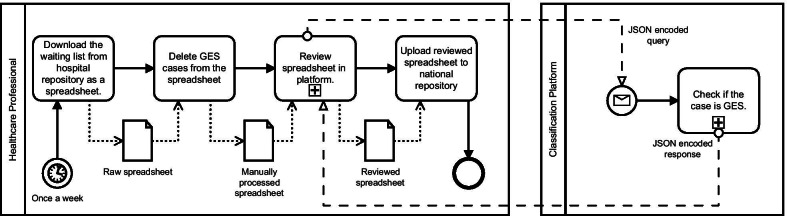
Diagram of the classification process. The input is the whole WL, and after removing the GES cases marked by the human, the non-GES WL is checked by the classification platform to make sure there are no GES cases in it, which should be prioritized by law. The panel on the left shows the frontend, while on the right is the backend

For implementing the classification models in the hospital, we designed a program that receives the whole waiting list (GES and non-GES cases), removes cases marked as GES by humans, and the non-GES are double-checked by our model (see Fig. 1). Please notice that we work this way since for the hospital it is much more hazardous a patient misclassified as non-GES and being GES than the other way around.

The backend was designed in Python using the Flask web framework. The service receives a JavaScript Object Notation (JSON) encoded message containing the referral information (diagnostic suspicion and patient's age). The message is then parsed, and the information is used as input in the model. In this process, the text data is preprocessed and vectorized, and the patient's age scaled. The model's predicted result is then compiled into another JSON message containing the Boolean result as a value of the GES key.

The frontend side of the deployment was developed in PHP, JavaScript, CSS, and HTML. A tailored web-based portal received an Excel spreadsheet of referrals containing the features needed to decide between GES and non-GES classes. The portal composes, parses, sends and receives JSON messages to display the results of the predictions in a user-friendly way.

The application checks every entry by sending requests to the Web service, and then displays, in a customized way, the human–machine discrepancies. Our application allows the user to discard or accept the classifier's suggestions. After managing discrepancies, the user can download rectified spreadsheet files.

A critical aspect of our application is that when conflicts are found, the platform retrieves the human-corrected class of each referral so the data can be later used to retrain the model. A general overview of the deployed platform is shown in Fig. 1.

The intended user profile for the classification platform is healthcare professionals in charge of uploading the waiting lists to the country-wide repository. These professionals should work with the platform for a final misclassification filter before uploading the rectified waiting list to the repository.

## Results

### Word embeddings

Word embeddings can be tested in two ways: intrinsically and extrinsically [39]. In the first case, two common tasks are the semantic analogy and the semantic similarity. For the extrinsic evaluation, on the other hand, we measure the performance on a downstream task that makes use of the embeddings [32, 33]. Since we were interested in choosing the best embedding for the GES/non-GES classification task, we selected between three different embeddings using this classification as extrinsic evaluation. We assessed the classification on the ground truth created by the three human experts.

The three embeddings were calculated using Word2vec with identical hyperparameters [24], but with different training corpora: the general dataset described previously (using non-GES referrals), the biomedical corpus [38], and the Spanish Billion Word Corpus, which captures the Spanish language in the general domain including Wikipedia [37]. As shown in Table 1, the general dataset, constructed with non-GES referrals, showed the best performance in the classification task. This result agrees with the work by Chen et al*.* [33] where they found, for the English language, that word embeddings trained over a clinical corpus outperform an embedding calculated over a general domain larger corpus.

**Table 1:** Extrinsic evaluation of Word2vec embeddings with identical hyperparameters, but different training corpora

Training corpus	Vocabulary size (tokens)	ROC AUC
General dataset	57,112	0.94
Biomedical literature	183,766	0.90
General Spanish language	1,000,653	0.90

### Development performance

The embedding with the best performance was used to vectorize the diagnostic suspicion, which was then used, along with the patient’s age, as input in machine learning classifiers. Table 2 summarized the performance of each machine learning model, where the hyperparameters were optimized via grid search. More details can be found in the GitHub repository that accompanies this publication,^1^ including the hyperparameter values in each case. The statistical significance of the difference between the mean performance of each of the combinations of models was significant, with a *p* value < 0.01.

**Table 2:** Performance of machine learning models

Model	ROC AUC (SD)
Logistic regression	0.91 (7.8 e-4)
Support vector machine	0.95 (5.4 e-4)
Random forest	0.96 (5.2 e-4)
Multilayer perceptron	0.95 (5.9 e-4)

Random Forest showed the best performance, reaching a ROC AUC of 0.96. Table 3 shows other metrics for this model for the GES, non-GES, and weighted by frequency classes for the testing dataset and the ground truth. The best hyperparameters for Random Forest, along with its hyperparameter grid, are shown in Table 3.

**Table 3:** Best hyperparameters for Random Forest along with its hyperparameter grid

Random forest		
Hyperparameter	Best value	Hyperparameter grid
Number of estimators	1600	200, 400, 600, 800, 1000, 1200, 1400, 1600, 1800, 2000
Minimum samples split	5	2, 5, 10
Max features	sqrt	sqrt, log2
Max depth	100	10, 20, 30, 40, 50, 60, 70, 80, 90, 100, 110
Bootstrap	True	True, false

A close inspection of Table 4 shows that the precision of the GES case in the training dataset is not very high (67%). Nevertheless, the recall is significantly better (90%). Possibly, the most critical metric in this case is the recall of the GES class, as we want to retrieve as many misclassified GES cases as possible. Having a high performance for the non-GES case (0.94 F1 in our model) is also essential if this system is to be used as a support for the clinical decision for a set of manually classified referrals.

**Table 4:** Performance of the Random Forest Classifier over the testing dataset and the ground truth constructed from human classifications

Class	Precision	Recall	F1-score	Number of examples
Testing dataset				
GES	0.67	0.90	0.77	37,502
non-GES	0.98	0.91	0.94	173,011
Weighted average	0.92	0.91	0.91	210,513
Ground truth dataset				
GES	0.92	0.55	0.69	260
No-GES	0.85	0.98	0.91	681
Weighted average	0.87	0.86	0.85	941

### Human–machine comparison

In order to compare the performance of the best method with a ground truth, we asked three health professionals related to WL classification to label 941 diagnostic suspicions as GES or non-GES. In 829 diagnoses, there were no discrepancies between the experts.

The experts’ agreement was further quantified using the Fleiss-Kappa coefficient, which is a statistical coefficient similar to Cohen's kappa but for more than two raters [40]. The three experts achieved 0.80 in the Fleiss-Kappa coefficient, which is considered a substantial agreement.

As shown in Table 5, the individual performance of humans is excellent. These raters were chosen to participate in this validation from their experience in the GES/non-GES classification, which is evidenced in these metrics.

**Table 5:** Expert performance over ground truth

Expert	Weighted average		
	Precision	Recall	F1-Score
1	0.96	0.96	0.96
2	0.95	0.94	0.94
3	0.95	0.95	0.95
Average	0.95	0.95	0.95

**Fig. 2:**
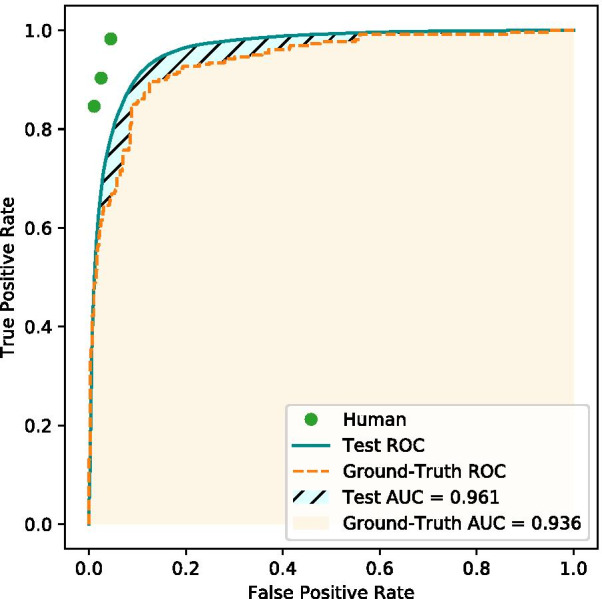
ROC curve for the human classification, best machine learning model in the testing dataset and over the ground truth dataset. Area under the curve (AUC) is also shown in the figure

Finally, the best machine learning classifier was tested on this ground truth dataset. Figure 2 displays the ROC curve.

**Fig. 3:**
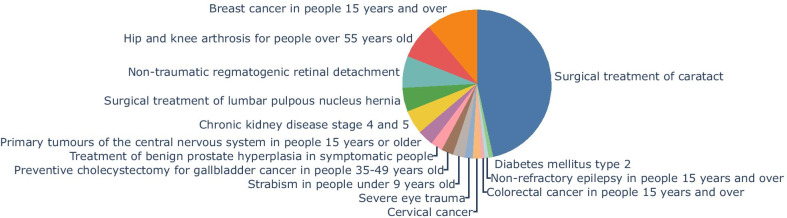
Distribution on non-detected GES cases (false negatives) in the ground-truth dataset

The predictions over the ground-truth dataset had a 13.5% of error, where 12.3% were false negatives and 1.2% were false positives. Figure 3 shows the distribution of false negatives. The majority of these cases arise from the surgical treatment of cataract, one of the most frequent health problems in the GES waiting list. This model mistake is not systematic, and it could be attributed to overfitting with the patient's age because this error is more frequent in some age bands.

The lowest metric in our models is the recall of the positive class in the GES task, which could not detect the total number of GES cases. However, we could detect the GES cases very precisely, lowering the number of false positives.

### Deployment

The Web application developed in this project is in agreement with the healthcare professional's workflow at Hospital Sótero del Rio. This professional is in charge of checking and uploading the non-GES WL to the National Repository, and our system works as a double-check for possible GES cases within. Additionally, in the deployed application, a chat with the development team was embedded so healthcare professionals can raise questions or comments regarding the platform. After seven months of consecutive and voluntary work, the platform analyzed 4,472 referrals. Human–machine discrepancies were 129 cases, wherein 87 (1.9%) cases the machine was right.

**Fig. 4:**
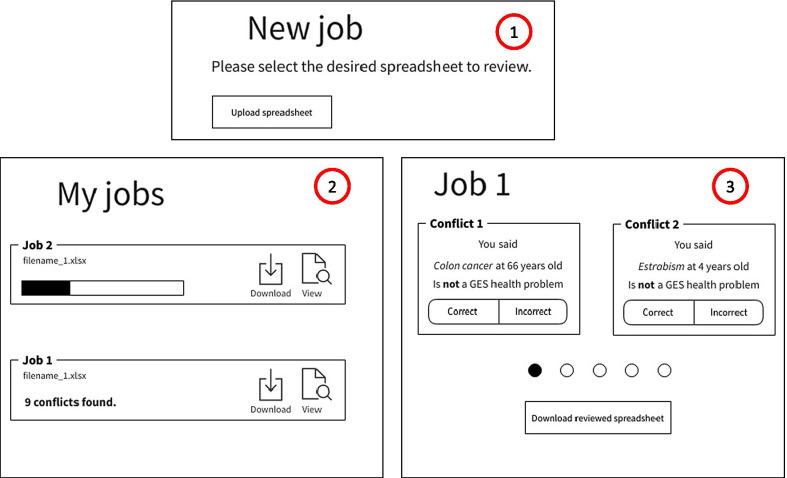
Wireframe representation of the platform. (1) Webpage to upload the spreadsheet in Microsoft Excel Format. This Excel contains both the GES and non-GES waiting lists. (2) Webpage showing the current spreadsheet being processed by the backend. (3) When conflicts are found, the user can manually solve each one by pressing if he/she is right, or the machine is right. At the end of this stage the user can download the corrected spreadsheet

A wireframe representation of the deployed web application is shown in Fig. 4.

## Discussion

The classifier and Web application reported in this paper is a result of nearly two years of collaboration between the SEMHS and the University of Chile. We wanted to identify a problem with clinical relevance, with enough data to train models, and where supporting the decision making could make a clear difference.

An automatic referral system that detects GES cases in the non-GES WL is beneficial at least in the following aspects: (a) it facilitates the job of the health professional in charge of the WL classification by supporting his/her decisions; (b) patients increase their chances of receiving prioritized attention when corresponds, and (c) hospitals avoid fines due to misclassifications. In summary, it provides support for better decision making, improving safety for patients and hospitals.

Patient misclassification can be detrimental to a patient's outcome. Waiting times in the non-GES WL are much longer than the GES WL, and a misclassification could even lead to a patient's death [41]. Furthermore, an increased waiting time directly affects the quality of life and social and psychological health of patients [54].

To classify free-text referrals, we first created a vector representation for each reason for referral and then used this vector in machine learning models. We used word embeddings to vectorize free-text narratives. For the work presented here, we did not use pre-defined embeddings for the general-domain Spanish language. We instead collected 11 million non-GES referrals for specialty consultations in the public healthcare system, obtaining vector representations tailored for this specific type of clinical narrative. The embeddings trained with this corpus showed the best performance in the classification task. Future work includes combining clinical, biomedical, and general datasets. We believe parameters, such as the size of the corpus to be added, should be considered as we do not want to lose an adequate representation of clinical jargon, which is vital in this type of application where the text entries are so abbreviated.

Word embeddings have been successfully used in the biomedical and clinical fields. Examples of applications in the Spanish language include the identification of entities in clinical narratives [34, 42], the expansion of abbreviations[43], the identification of negation[44], or for the automatic codification of diseases [45], to mention some.

For the classification of referrals in GES and non-GES classes, we used a variety of machine learning models. They received as input the vectorization of the referral as well as the age of the patient. The use of machine learning in medicine has been slower than in other disciplines, but its extensive use is auspicious [46]. We can roughly group machine learning applications in those using *classical* machine learning methods and those that use *deep* learning. A key aspect of choosing between them is the amount of training data, processing power and if interpretation of the models is a must [47]. In our case, from the amount of data and processing power we had, classical machine learning methods were the most suitable option.

In terms of the model's explainability, we did not experience any concern from the team at the hospital since the system was designed as a double-check for the human in charge, and this person has the final decision. In other words, there were no worries about the model changing people from waiting lists without understanding what features were used for classification, as in case of conflict, the human expert could decide to take or not the suggestion.

For our application, the method with the best performance was Random Forest, which has been widely used since its publication in 2001 [48]. Its use in medicine has been natural as, it can be explained as an arrangement of decision trees, a concept rooted in medicine. In terms of medical applications, we find various successful cases of the application of tree-based methods. Examples include the detection of hospital-acquired infections [49], the prediction of obesity rates from food sales [50], or detecting suicidal behavior in emergency consultations [51].

Our model achieved a weighted average F1-Score metric of 0.85, calculated over an independent ground truth dataset labeled by three medical experts in the field of waiting lists. In the classification task, humans achieved a substantial agreement, which reflects the expertise of the professionals selected.

Differences between the reported performance in testing and validating phases can be explained by moderate overfitting in the training dataset. To lower the overfitting, we can get more training data by using the platform in another hospital or using another balancing method for the training subset, such as upsampling the minority class using the Synthetic Minority Oversampling Technique [52]. The lowest metric in our models is the recall of the positive class when tested against the ground truth. This means we were detecting more GES cases than the actual ones. Nevertheless, these false negatives were not overloading the human revision. Our primary goal was to detect the GES cases very precisely, even at the cost of a relatively low recall.

In terms of time used by the machine vs. humans, the automatic classification takes 10 min in a daily-usage laptop to predict the class of 1000 referrals. In contrast, each human took around 120 min to label the same 1000 referrals. Therefore, even if the automatic classifier does not outperform human experts, our method is significantly faster than human labeling. On top of that, the amount of highly qualified health professionals is heterogenous along the country. Considering that humans roughly misclassify non-GES cases 15% of the time (85% recall of the negative class), our system outperforms this metric, lowering patient misclassification.

In order to enhance the performance of the classifier, we hope to deploy the platform in other hospitals or, even better, automatically check the non-GES WL in the National Repository in the Ministry of Health. A second way to improve our work is to retrain the model by taking into account the machine's mistakes compared to the health professional in charge of WL and the comparison with the ground truth. Due to the large number of examples used in the training process, we could create synthetic examples of these mistakes to enforce the learning over these cases. Moreover, under a scenario where significantly more data is available, we could use more advanced machine learning methods to solve this task, such as Recurrent Neural Networks with attention mechanisms [53], state of the art in predicting over free-text inputs.

In recent years, pre-training strategies have become state-of-the-art in many NLP tasks. In particular, the BERT model [53]. Thus, it is reasonable to ask how BERT would work in our problem. Several aspects prevented us from using BERT. Firstly, there was no BERT model for the Spanish Language at the moment of the platform's deployment. Now, there is a version for Spanish [54], but a significant number of words in our vocabulary are not in the Spanish BERT vocabulary. BERT uses subwords to handle out-of-vocabulary tokens, but more than 37% of the words in our corpus (that is, 1,366,657 words) need two or more tokens to be represented in the Spanish BERT, which would lead to suboptimal results. Moreover, the lack of sufficient computing power in the hospitals' servers was a key issue preventing us from using BERT as computing BERT embeddings in CPU is two orders of magnitude slower than a classical embedding look-up table (as in the case of our current solution). This also ruled out a BERT fine-tuning approach.

The team established a close collaborative relationship with the WL team throughout the entire project duration. Before developing the model, we collaborated in several data cleansing tasks and classified several thousands of referrals using keywords in SQL queries. Based on that experience, exploring advanced methods to optimize classification seemed reasonable to the team. When we discovered the high performance of the model, implementing a web application was the natural step. The user interface was designed in close collaboration with the clinical team. We referred to the system as “Nur-i” to recognize their participation (the responsible for WL classification and loading was named Nury). During the implementation, we closely monitored their reaction and made the necessary adjustments. In the end, they felt it was their system.

Our solution does not replace human decisions; instead, it provides a second opinion based on historical information. The voluntary and continuous use for seven months demonstrates its usefulness for the healthcare professional that used the platform. This person did not need considerable extra time or specialized training to use the classifier, which was a crucial factor in the success of this project. Detailed understanding of the WL reporting process and an agile development approach were crucial for deploying the application in a large hospital correctly. Besides, in this project, we verified what the literature states: successful machine learning projects in healthcare require both clinical and technical participants with solutions that can be used following the clinical workflow [55].

## Conclusions

We were able to deploy a production-ready system to automatically classify referrals into GES and no-GES in a public hospital in Chile. The performance of the platform was compared with a ground truth made from the classification of three waiting list experts, and the automatic system is moderately worse than human classification, but more than ten times faster than the experts.

In order to use the information contained in the reason for referrals, we used neural word embeddings specifically trained over Chilean clinical text. These vectors were the input of machine learning algorithms that classify diagnoses into GES and non-GES categories, with Random Forest showing the best performance. The platform was tailored to be adapted to the current data-cleaning workflow of the healthcare professional, used continuously and voluntarily in the system for seven months.

The use of our system is helping Chile achieve the healthcare objectives of the decade [56] because (a) we are improving the quality of the health information systems by erasing human error in their records, (b) empowering cross-sector research by implementing computer science elements into the public healthcare sector, (c) improving the quality of sanitary technologies by applying cutting-edge methods to their information infrastructure and (d) improving patient satisfaction by decreasing misclassification and waiting times for GES patients.

## Data Availability

The word embeddings trained over 11 million free text diagnostics from all over Chile are shared in 10.5281/zenodo.3924799 and the ground truth can be found here https://zenodo.org/record/4737751. The code to reproduce all the results presented in this article is https://github.com/fvillena/referral_classifier.

## Abbreviations

GES: Explicit Health Guarantees (acronym in Spanish)
WL: Waiting list
SEMHS: South East Metropolitan Health Service
TF-IDF: Term Frequency-Inverse Document Frequency
ROC AUC: Area under the curve of the receiver operating characteristic
JSON: JavaScript Object Notation
SD: Standard deviation

## Footnotes

1 https://github.com/fvillena/referral_classifier.
