# Correction to: Supporting the classifcation of patients in public hospitals in Chile by designing, deploying and validating a system based on natural language processing

**DOI:** 10.1186/s12911-021-01587-7

**Published:** 2021-07-20

**Authors:** Fabián Villena, Jorge Pérez, René Lagos, Jocelyn Dunstan

**Affiliations:** 1 Center for Mathematical Modeling - CNRS UMI2807, Faculty of Physical and Mathematical Sciences, University of Chile, Santiago, Chile; 2 Center for Medical Informatics and Telemedicine, ICBM, Faculty of Medicine, University of Chile, Santiago, Chile; 3 Computer Science Department, Faculty of Physical and Mathematical Sciences, University of Chile, Santiago, Chile; 4 Millennium Institute for Foundational Research on Data, Santiago, Chile; 5 Digital Health Unit, South East Metropolitan Health Service, Santiago, Chile

## Correction to: BMC Med Inform Decis Mak 21:208 (2021) 10.1186/s12911-021-01565-z

Following publication of the original article [1], it was reported that the list of authors was published in the incorrect order. Jocelyn Dunstan was erroneously listed as the first author instead of the last. The authorship order has been amended and the original article has been updated.

## References

[1] Villena F, Pérez J, Lagos R (2021). Supporting the classification of patients in public hospitals in Chile by designing, deploying and validating a system based on natural language processing. BMC Med Inform Decis Mak.

